# Family digital learning support and students’ self-regulated learning in flipped classrooms: the roles of home–school learning responsibility reconfiguration, responsibility internalization, and digital learning self-efficacy

**DOI:** 10.3389/fpsyg.2026.1786428

**Published:** 2026-06-03

**Authors:** Jiaohui Tang, Shuqing Zhan, Baojian Wei

**Affiliations:** 1 Department of Education, Graduate School, Kookmin University, Seoul, Republic of Korea; 2 Faculty of Education, Yunnan Normal University, Kunming, Yunnan, China; 3 School of Nursing, Shandong First Medical University & Shandong Academy of Medical Sciences, Jinan, Shandong, China

**Keywords:** digital learning self-efficacy, family digital learning support, flipped classroom, home–school learning responsibility reconfiguration, responsibility internalization, self-regulated learning

## Abstract

As flipped-classroom teaching becomes increasingly common in university courses, more learning preparation now takes place outside the classroom in digital environments, where family resources may shape the conditions under which learning occurs. Using a sample of university students enrolled in flipped-classroom courses, this study examines how family digital learning support relates to self-regulated learning. The analytical framework considers the roles of family digital learning support, home–school learning responsibility reconfiguration, responsibility internalization, and digital learning self-efficacy in shaping students’ learning management in digitally mediated contexts. The results show that family digital learning support is positively associated with self-regulated learning and remains significant after demographic variables are controlled (B = 0.504, *p* < 0.001). In the baseline model, family digital learning support (B = 0.248, *p* < 0.001), responsibility internalization (B = 0.353, *p* < 0.001), and home–school learning responsibility reconfiguration (B = 0.220, *p* < 0.001) are all positively associated with self-regulated learning. In the full model, family digital learning support (B = 0.277, *p* < 0.001), home–school learning responsibility reconfiguration (B = 0.186, *p* < 0.001), responsibility internalization (B = 0.347, *p* < 0.001), and digital learning self-efficacy (B = 0.155, *p* < 0.001) remain significant predictors. Bootstrap mediation analysis identified a small but statistically significant negative indirect effect through digital learning self-efficacy (indirect effect = −0.018, 95% CI [−0.038, −0.001]), which should be interpreted cautiously because of its small magnitude and theoretically unexpected direction. Moderation analysis showed no significant group-based interaction effects, suggesting that these relationships remain broadly stable across groups. Overall, the findings indicate that self-regulated learning in flipped-classroom contexts is closely associated with family digital learning conditions and responsibility-related learning processes, while the indirect role of digital learning self-efficacy appears limited.

## 1 Introduction

### 1.1 Flipped-classroom learning in higher education and the growing involvement of families in digital study support

In many university courses, flipped classrooms have moved from experimental teaching approaches to regular instructional arrangements. As blended and online learning formats have become more common across higher education, pre-class preparation is increasingly completed on digital platforms, while classroom time is used for discussion, collaboration, and applied problem-solving. Students’ learning activities are therefore distributed across campus and home settings rather than concentrated solely in physical classrooms. Kim et al. (2021) reported that in university flipped-classroom courses, stable instructional routines and sustained student participation form part of everyday teaching practice, suggesting that flipped classrooms are now integrated into ordinary course delivery rather than treated as short-term innovations. Research has consequently paid growing attention to students’ learning behaviors in out-of-class digital environments. Shih et al. (2019) found that university students in flipped classrooms rely on online learning platforms to complete pre-class tasks, making self-regulated learning an important factor in academic performance. Under these conditions, learning responsibility extends beyond classroom supervision, requiring students to plan study time and monitor learning progress on their own. Jeong and González-Gómez (2025) further showed that flipped and gamified flipped classrooms are implemented across university courses in multiple countries and are associated with comparable effects on students’ learning attitudes and emotional engagement. In English as a Foreign Language (EFL) learning contexts, flipped classrooms have also been widely implemented to support language practice and independent learning outside the classroom. These findings suggest that flipped classrooms have become a widely used instructional format rather than a practice limited to specific institutions or disciplines. Importantly, this pattern also extends to Confucian-heritage educational contexts, where the adoption of flipped learning may interact with culturally rooted expectations about teaching, participation, and authority. For example, Karjanto and Simon (2019) examined English-medium instruction in a Confucian-heritage culture and showed that flipped classroom implementation raises not only pedagogical questions but also cultural ones regarding how learning practices align with established classroom norms. As digital tools are more deeply incorporated into university teaching, learning activities conducted outside the classroom leave increasing digital records. Using survey and learning-analytics data, Han (2025) showed that self-regulated learning processes in university flipped classrooms can be observed and quantitatively examined, reflecting the growing role of data in supporting learning management. In this setting, students’ out-of-class learning behaviors depend not only on personal motivation but also on practical conditions such as home learning environments, access to digital devices, and daily time arrangements. Flipped classrooms are also often combined with project-based learning, which places greater demands on students’ task engagement outside class. Köpeczi-Bócz (2024) reported that university courses integrating flipped and project-based learning enhance learning motivation while increasing requirements for study time management and sustained participation beyond the classroom. Overall, a substantial share of learning in flipped-classroom settings now takes place outside the university, and family-provided digital facilities, internet access, and home study spaces form important conditions supporting this work. In this sense, family resources have regained relevance in students’ higher-education learning processes.

### 1.2 Changing patterns of home–school learning responsibility in digital and flipped learning environments

In university teaching contexts where digital instruction has expanded and flipped classrooms are routinely adopted, questions about how learning responsibility is shared among schools, students, and families have attracted increasing scholarly attention. Early flipped-classroom research focused on classroom reorganization, highlighting students’ greater involvement in pre-class preparation and in-class participation. Urquiza-Fuentes (2020) reported that partial adoption of flipped classrooms can strengthen students’ responsibility-taking and improve self-regulated learning, and related studies similarly suggest that flipped classrooms can promote more active learning management and preparation behaviors (Purba, 2025; Nurwulandari, 2024). As learning activities increasingly take place beyond the classroom, responsibility has come to be understood not only in terms of student engagement but also as a shared arrangement involving schools, students, and families. In this context, family conditions have entered responsibility discussions more directly. Azhari et al. (2023) showed that family support, learning motivation, and self-regulated learning are jointly related to learning engagement, while Dayimani and Padayachee (2023) found that self-regulation-based strategies in flipped computer-programming courses reshape participation patterns, with a greater share of study occurring outside the classroom. Du et al. (2023) further showed that direct and indirect recommendations in flipped online activities can facilitate students’ self-regulated learning, reinforcing the importance of out-of-class support structures. Subsequent work has linked responsibility more closely to instructional design decisions: when substantial learning tasks are shifted to digital environments beyond class time, students must assume greater responsibility for managing time, coordinating resources, and monitoring progress. Recent studies likewise suggest that flipped classrooms can strengthen students’ digital fluency, self-directed learning, and broader self-management capacities (Elhilal, 2025; Zhong, 2024). More recent research has turned to how responsibility transfer can be sustained. Tromp (2025) argued that scaffolded course design supports learning in flipped classrooms, while van Alten et al. (2020) found that support for self-regulated learning embedded in flipped learning videos can enhance students’ learning processes. Cabı and Türkoğlu (2025) likewise showed that a learning analytics–based feedback system in a flipped classroom can improve both academic achievement and self-regulated learning. These studies suggest that responsibility transfer is more sustainable when students receive structured support rather than being left to manage digital learning demands alone. Research on family involvement has also become more explicit. Zhang et al. (2025) reported that parental educational involvement influences self-regulated learning through students’ learning attitudes. Although based on secondary-school samples, this work offers useful insight into how family participation may operate through psychological and behavioral channels, providing direction for examining family digital support and responsibility arrangements in higher-education settings. Taken together, existing studies point to a progression from research on responsibility within flipped classrooms, to attention to out-of-class learning settings, and further to consideration of family and technological conditions supporting sustained responsibility-taking. Yet how family digital learning support and home–school responsibility restructuring interact within a unified explanatory framework remains insufficiently examined in higher-education contexts.

### 1.3 Responsibility internalization and digital learning self-efficacy in university students’ self-regulated learning

Early work on self-regulated learning has shown that students’ willingness to accept and take ownership of learning responsibilities plays an important role in the long-term development of self-regulated learning. Perry and VandeKamp (2000) observed that classrooms supporting self-regulated learning typically clarify learning expectations, allow room for autonomy, and encourage reflection. Under such conditions, tasks assigned by teachers can gradually be taken up by students as responsibilities they recognize as their own. This line of research established responsibility internalization as a key process in self-regulated learning. As flipped classrooms have been adopted in higher-education teaching, the organization of learning across time and space has changed, and pre-class and out-of-class digital study has become part of routine course arrangements. Onodipe et al. (2020) reported that learning management systems in flipped classrooms provide instructional materials and assist students in planning and tracking their study through records, reminders, and feedback. Their results suggest that in digital flipped-classroom settings, responsibility internalization is shaped not only through in-class interaction but also through repeated participation in online learning activities. Psychological experiences in digital learning environments also influence how consistently students apply self-regulated learning strategies. Hwang (2025) found that students’ background characteristics are related to differences in self-regulated learning performance in flipped classrooms, indicating that identical instructional designs do not lead all students to engage or apply strategies in the same way. This variation points to the potential importance of both responsibility internalization and students’ confidence in digital learning situations for explaining differences in self-regulated learning. Recent studies have further highlighted digital learning self-efficacy in this process. Han et al. (2025) showed that self-regulated learning strategy training embedded in pre-class digital activities in flipped classrooms increases students’ learning self-efficacy and reduces anxiety. Their findings indicate that confidence in managing digital learning tasks supports continued engagement and the use of learning strategies in online study. Although prior research has examined responsibility internalization, self-efficacy, and self-regulated learning from different perspectives, these elements are often studied separately in higher-education flipped-classroom contexts. In flipped classrooms, substantial portions of university learning now take place in out-of-class digital environments, bringing family learning spaces and home digital conditions into students’ study routines. How family digital learning support influences students’ acceptance of learning responsibility, how digital learning self-efficacy develops in this process, and how these factors work together to sustain self-regulated learning remain open questions. Examining these relationships within a unified analytical framework can deepen understanding of how learning responsibility is taken up in flipped-classroom contexts and clarify how digital learning conditions shape self-regulated learning in university settings.

## 2 Review

### 2.1 Family digital learning support in flipped-classroom contexts in higher education

In many higher-education courses, flipped classrooms now operate as a regular teaching format, with digital platforms and online resources supporting a substantial share of learning activities. Learning support therefore extends beyond classroom interaction into out-of-class digital study spaces. Anyichie et al. (2023) reported that supportive learning environments influence students’ engagement and self-regulation, and that alignment between instructional design and learning settings contributes to continued participation. Their findings show that in digital flipped-classroom contexts, learning support involves not only teacher guidance but also the external environments where students carry out their study. Regarding digital infrastructure, Fiqri et al. (2024) found that flipped-classroom models built on digital self-learning networks improve students’ organization of learning and autonomous study capacity. They noted that the reliable operation of digital platforms plays an important role in course implementation. These results point to the importance of adequate technological equipment and stable internet access for completing out-of-class digital learning tasks. In flipped classrooms, home settings often serve as the main location for such study. Purba (2021) showed that in home-based learning, digital media and online platforms place learning activities within everyday household routines, and that family-provided devices, internet access, and suitable study spaces influence the continuity and quality of out-of-class work. In course designs that rely heavily on digital pre-class learning, families therefore contribute directly to students’ ability to complete preparatory tasks. From the perspective of learning experience, Bolandifar and Salehi (2024) observed that flipped classrooms in undergraduate online learning affect students’ learning anxiety and willingness to participate, and that emotional experiences during digital study are linked to support available in the learning environment. Wang (2023) likewise reported that online collaborative flipped classrooms enhance self-regulated learning, with online interaction structures and organization of out-of-class study supporting learning processes. As more courses adopt online or blended flipped formats, researchers have paid increasing attention to students’ strategy use during out-of-class learning. Diningrat et al. (2024) found that in online flipped classrooms, self-regulated learning strategies are associated with patterns of digital platform use, suggesting that digital support systems shape how students organize their study activities. Recent research has also highlighted digital literacy as an important background factor in flipped-classroom participation. Moundy (2025) showed that digital literacy and digital soft skills improve students’ adaptability and learning efficiency in digital environments, and that these competencies develop through study in home and personal learning spaces. Across these studies, evidence consistently shows that in higher-education flipped-classroom settings, digital learning support plays a central role in students’ out-of-class study. Family-provided devices, network access, and home study spaces support students in completing pre-class learning tasks, offering a basis for examining how family digital learning support relates to learning responsibility.

### 2.2 Home–school learning responsibility reconfiguration in flipped learning

Flipped classrooms reorganize how learning tasks are arranged in university courses by moving knowledge acquisition and initial understanding to digital study conducted outside class. As a result, a larger share of learning work is completed by students independently rather than under continuous in-class guidance. Zheng and Zhang (2020) reported that in medical flipped-classroom courses, self-regulated learning is closely linked to academic performance. When classroom sessions focus on discussion and application, students must complete preparatory learning tasks before class for the course to function as intended. Altas and Mede (2020) likewise found that flipped instruction improves pre-service teachers’ writing performance and self-regulated learning, alongside greater reliance on out-of-class task completion. Jin and Harp (2020) observed that in flipped teacher-education courses, students’ self-efficacy and capacity to integrate technology relate to how they perceive teamwork and task responsibility. Their findings show that flipped instruction changes both teaching practices and students’ perceptions of their own role in learning. Studies at other educational stages report similar patterns. Makinde (2020) showed that secondary-school mathematics students in flipped classrooms achieve higher academic outcomes while completing more preparation and practice outside class. Research has therefore begun to examine how students manage learning responsibilities across different study settings. Lee et al. (2023) found that self-regulated learning interventions strengthen primary students’ learning management skills, indicating that responsibility-taking develops through guided learning experiences rather than appearing automatically. Mukminin and Kusumasari (2025) reported that instructional media design supports junior high school students’ self-regulated learning. Their results illustrate that when learning activities take place in everyday and home settings, responsibility-taking depends in part on support provided outside school. University-level research reports comparable observations. Odhiambo (2025) found that flipped classrooms in Kenyan universities produce stronger academic outcomes than traditional classrooms, accompanied by sustained engagement in study tasks beyond class time. In such courses, much of students’ learning activity occurs in personal study environments, which often overlap with family spaces. Across existing studies, flipped instruction is consistently associated with the movement of learning tasks beyond classroom boundaries and with greater student involvement in preparation, task completion, and progress monitoring outside school. These patterns provide a basis for examining how family digital learning support relates to students’ learning responsibility in flipped-classroom settings.

### 2.3 Responsibility internalization, digital learning self-efficacy, and self-regulated learning in flipped-classroom contexts

In flipped-classroom courses, self-regulated learning develops through both instructional arrangements and students’ own acceptance of learning responsibility, together with their confidence in handling digital learning tasks. When students take an active role in managing out-of-class study tasks, responsibility for learning shifts from externally assigned requirements toward student-initiated action. In this sense, self-regulated learning can be understood not only as an outcome of effective flipped-classroom design but also as a process supported by responsibility internalization and digital learning self-efficacy. Alkhalaf (2023) reported that introducing self-regulated learning strategies in EFL flipped classrooms improves students’ grammar performance, suggesting that structured support can strengthen students’ ability to manage their own learning. Samaila et al. (2024) found that a think–pair–share flipped-classroom model improves academic achievement and strengthens students’ learning self-efficacy, indicating that confidence in completing digital learning tasks supports continued engagement in study activities. Tsai et al. (2025) further observed that mobile-supported flipped language learning enhances learning motivation, learning behaviors, and academic performance, pointing to the combined influence of digital learning confidence and responsibility-taking in shaping self-regulated learning. Phung et al. (2025) likewise reported that flipped instruction strengthens students’ English learning motivation and self-regulated learning, alongside stable learning confidence and sustained goal commitment during digital learning tasks. Evidence from other educational stages shows comparable patterns. Permitasari et al. (2025) found that academic flow and self-efficacy are strongly related to self-regulated learning, suggesting that learning confidence supports responsibility-taking across developmental stages. Chen and Hung (2025) showed that integrating self-regulated learning strategies into flipped nursing courses improves students’ skill acquisition and self-management, illustrating that instructional design can strengthen both responsibility-taking and digital learning confidence in support of self-regulated learning. Li (2024) further reported that delayed gratification and psychological resilience contribute to self-regulated learning, indicating that persistence in learning tasks is accompanied by the internal acceptance of learning responsibility, particularly in digital learning contexts. Taken together, prior research consistently suggests that in flipped classrooms relying heavily on out-of-class digital study, self-regulated learning is sustained not only by instructional design but also by students’ internalization of learning responsibility and their confidence in managing digital learning tasks. Building on this evidence, the present study examines how family digital learning support relates to university students’ self-regulated learning, with particular attention to the roles of responsibility internalization and digital learning self-efficacy in higher-education flipped-classroom settings.

## 3 Methods

### 3.1 Participants

This study employed a cross-sectional survey design to examine how family digital learning support relates to students’ self-regulated learning in flipped-classroom contexts, with particular attention to the role of home–school learning responsibility reconfiguration and digital learning self-efficacy. The target population consisted of undergraduate students enrolled in university courses that adopted flipped-classroom teaching approaches. In these courses, students are typically required to complete pre-class learning tasks through digital platforms and engage in discussion and application activities during classroom sessions. Because a substantial portion of learning takes place outside the classroom, students’ access to digital learning resources and study conditions in their home environments becomes an important factor shaping their learning experiences. Data were collected through an online questionnaire distributed to students during the academic semester. A total of 650 questionnaires were distributed, and 595 responses were returned, yielding a response rate of 91.5%, indicating strong participation from the target population. Prior to statistical analysis, the dataset was carefully screened to ensure data quality. Responses were excluded if they met one of the following criteria: (1) incomplete questionnaires with substantial missing responses, (2) response patterns indicating straight-lining across scale items, or (3) extremely short completion times suggesting inattentive or invalid responses. After applying these screening procedures, 45 questionnaires were removed, resulting in a final analytical sample of 550 valid responses.

The participants ranged in age from 18 to 23 years (M = 20.28, SD = 1.25), which is consistent with the typical age distribution of undergraduate students. In terms of gender composition, 298 participants were male (54.2%) and 252 were female (45.8%). For analytical purposes, respondents were also categorized into two groups according to the grouping variable used in the model, with 265 participants (48.2%) in Group 0 and 285 participants (51.8%) in Group 1. The final sample size exceeds commonly recommended thresholds for regression and mediation analyses in behavioral and educational research and therefore provides adequate statistical power for detecting meaningful relationships among the study variables. The demographic characteristics of the sample are presented in Tables 1, 2.

**Table 1:** Demographic characteristics of the sample (*N* = 550): gender and group membership reported as frequencies and percentages.

Variable	Category	*N*	%
Gender	Male	298	54.2
Female	252	45.8	
Group	Group 0	265	48.2
Group 1	285	51.8	

**Table 2:** Descriptive statistics for participant age (*N* = 550), ranging from 18 to 23 years.

Variable	Mean	SD	Min	Max
Age	20.282	1.251	18	23

### 3.2 Measures

All constructs in this study were measured using multi-item Likert-type scales, with responses rated on a five-point scale ranging from 1 (strongly disagree) to 5 (strongly agree). The questionnaire items were adapted from established instruments in educational psychology and digital learning research and were revised to fit the context of flipped-classroom learning in higher education. Several constructs were conceptually informed by widely used theoretical frameworks related to self-regulated learning and self-efficacy (e.g., Bandura, 1997; Zimmerman, 2002; Pintrich, 2004), and the measurement items were adjusted to reflect learning behaviors in digitally mediated and flipped-classroom environments. Family digital learning support (FDLS) captures the extent to which students receive digital learning resources and environmental support within their family environments, including access to digital devices, stable internet connectivity, and supportive home learning conditions that facilitate out-of-class study in flipped classrooms. This construct was measured using six items, with example items including “My family provides stable internet access that supports my online learning” and “I have access to digital devices at home that allow me to complete online learning tasks effectively.” home–school learning responsibility reconfiguration (HSLRR) reflects students’ perception that learning responsibilities in flipped classrooms are increasingly distributed between classroom instruction and independent study outside the classroom, capturing the extent to which students recognize that they must assume greater responsibility for managing learning tasks beyond direct teacher supervision. It was measured using five items, with example items including “In this course, I need to take responsibility for preparing learning materials before class” and “A large part of my learning progress depends on how I organize my study outside the classroom.” responsibility internalization (RI) refers to the extent to which students internalize learning responsibilities as personal obligations rather than externally imposed tasks, reflecting the degree to which students actively accept responsibility for managing their own learning processes. This construct was measured using five items, with example items including “I feel personally responsible for completing the learning tasks required in this course” and “I see course learning tasks as obligations that I should manage on my own.”

digital learning self-efficacy (DLSE) represents students’ confidence in their ability to complete learning activities in digital environments and effectively manage online learning tasks, capturing their perceived competence in navigating digital learning platforms, organizing online study activities, and solving problems encountered during digital learning. It was measured using four items, with example items including “I feel confident in my ability to complete learning tasks using online learning platforms” and “I can effectively manage my learning activities when studying in digital environments.” self-regulated learning (SRL) represents students’ perceived ability to plan, monitor, and regulate their learning processes during course participation, including organizing study schedules, monitoring learning progress, and adjusting strategies when encountering difficulties. It was measured using six items, with example items including “I am able to plan my learning activities effectively when studying course materials” and “I can adjust my learning strategies when I encounter difficulties during study.” In addition to demographic variables such as age and gender, a grouping variable was included in the analysis to examine whether the relationships among the core constructs differed across student subgroups. The grouping variable divided respondents into two categories (Group 0 and Group 1) based on differences in learning context characteristics reported in the survey, and it was used in the moderation analysis to explore potential variation in the model relationships across student groups. To assess the quality of the measurement instruments, internal consistency reliability and convergent validity analyses were conducted for all constructs. The corresponding results, including Cronbach’s alpha coefficients, composite reliability (CR), and average variance extracted (AVE), are reported in the Results section. Overall, most constructs demonstrated acceptable to good internal consistency, supporting their use in the subsequent correlation, regression, and mediation analyses.

### 3.3 Procedure

This study adopts an explanatory quantitative approach to examine out-of-class digital learning in flipped-classroom contexts in higher education. The focus is on how family digital learning support influences university students’ self-regulated learning through responsibility-related processes. The data were derived from a cross-sectional questionnaire survey. In the participating courses, the flipped-classroom model was implemented throughout the semester. Students were typically required to complete pre-class learning tasks through the university’s Massive Open Online Course (MOOC) platform, including watching instructional videos, reading digital learning materials, and completing short quizzes designed to ensure basic understanding before entering classroom discussions. Classroom time was then primarily devoted to discussion, collaborative activities, and problem-solving tasks. This instructional arrangement means that a substantial portion of learning preparation takes place outside the classroom through the MOOC platform. The analytical model follows a sequence from learning conditions to psychological processes and finally to learning outcomes. Family digital learning support represents the primary external condition, while self-regulated learning is the main outcome. Two psychological processes are examined: responsibility internalization and digital learning self-efficacy. In addition, home–school learning responsibility reconfiguration is included to capture changes in the distribution of learning responsibility under flipped-classroom arrangements. The model examines both direct associations and mediating pathways in order to clarify how external support conditions influence students’ self-management of learning through responsibility-related mechanisms. The statistical procedures were aligned with the research questions. First, the internal consistency of the multi-item measures was assessed. Descriptive statistics and correlation analyses were then conducted. Subsequently, regression models and bootstrapping procedures were used to test mediation effects. Multicollinearity diagnostics were performed, and robustness checks were conducted using standardized variables. Interaction terms were introduced to examine whether key relationships differed across student groups. These steps allow the proposed associations to be evaluated in a consistent and transparent manner.

### 3.4 Data analysis

Data analysis was conducted using Python statistical analysis libraries to examine the relationships among FDLS, HSLRR, RI, DLSE, and SRL. The analytical procedure consisted of several sequential stages designed to assess both measurement quality and the associations among the study variables. First, descriptive statistics were calculated to summarize the distributional properties of the variables, including means, standard deviations, minimum and maximum values, skewness, and kurtosis (Table 3). Second, the reliability and construct validity of the measurement scales were evaluated through internal consistency and convergent validity analyses using Cronbach’s alpha, CR, and AVE (Table 4). Third, Pearson correlation analysis was conducted to examine the zero-order relationships among the core variables in the study (Table 5). To assess potential common method bias, Harman’s single-factor test was performed by examining the variance explained by the first unrotated factor in an exploratory factor analysis (Table 6).

**Table 3:** Descriptive statistics for the five core study variables (*N* = 550).

Variable	Mean	SD	Min	Max	Skew	Kurtosis
Family digital learning support	2.993	0.694	1.000	5.000	0.038	−0.215
Home–school learning responsibility reconfiguration	2.990	0.723	1.200	5.000	−0.074	−0.321
Self-regulated learning	2.989	0.709	1.000	4.667	−0.061	−0.312
Responsibility internalization	2.999	0.693	1.000	4.800	0.061	−0.309
Digital learning self-efficacy	3.004	0.667	1.000	4.750	−0.047	−0.111

All items used a five-point Likert scale. Skewness and kurtosis values indicate approximately normal distributions.

**Table 4:** Internal consistency (α), composite reliability (CR), average variance extracted (AVE), and factor loading ranges for all constructs (*N* = 550).

Construct	Items	Cronbach’s α	CR	AVE	CFA unstandardized loading range	EFA loading range
Family digital learning support	6	0.870	0.891	0.578	0.892–0.975	0.664–0.762
Home–school learning responsibility reconfiguration	5	0.863	0.886	0.608	0.912–1.067	0.643–0.738
Self-regulated learning	6	0.886	0.914	0.640	0.958–1.006	0.659–0.797
Responsibility internalization	5	0.819	0.851	0.533	0.980–1.117	0.699–0.767
Digital learning self-efficacy	4	0.714	0.714	0.384	1.000–1.144	0.559–0.664

Recommended thresholds: α > 0.70, CR >0.70, AVE >0.50. CR, composite reliability; AVE, average variance extracted. The CFA loading range reported here refers to unstandardized factor loadings. Global model fit: χ^2^(292) = 411.603, χ^2^/df = 1.41, CFI = 0.980, TLI = 0.977, RMSEA = 0.027 (90% CI [0.021, 0.033]).

**Table 5:** Zero-order Pearson correlations among FDLS, HSLRR, SRL, RI, and DLSE (*N* = 550).

Variable	Family digital learning support	Home–school learning responsibility reconfiguration	Self-regulated learning	Responsibility internalization	Digital learning self-efficacy
Family digital learning support	1				
Home–school learning responsibility reconfiguration	0.501***	1			
Self-regulated learning	0.495***	0.561***	1		
Responsibility internalization	0.324***	0.365***	0.421***	1	
Digital learning self-efficacy	−0.085**	−0.030 ns	0.161*	0.151*	1

*p < 0.05, **p < 0.01, ***p < 0.001; ns, not significant.

**Table 6:** Harman’s single-factor test results based on unrotated exploratory factor analysis (*N* = 550).

Factor	Variance explained (%)
Factor 1	31.046
Factor 2	10.506
Factor 3	7.584
Factor 4	6.315
Factor 5	5.572

The first factor explained 31.05% of total variance, below the 40% threshold, suggesting common method bias is not a serious concern.

Subsequently, multiple regression analyses were conducted to examine the direct association between FDLS and SRL while controlling for demographic variables (Table 7). A baseline regression model was then estimated by including FDLS, RI, and HSLRR as predictors of SRL (Table 8). Next, a full regression model was estimated by additionally including DLSE to examine its association with SRL alongside the other predictors (Table 9). To further examine indirect relationships, mediation analysis was conducted using a bootstrap resampling procedure with 1,500 samples to estimate the indirect effect of FDLS on SRL through DLSE and the corresponding confidence intervals (Table 10). Finally, moderation analysis was performed by introducing interaction terms involving FDLS, RI, and group membership to examine whether the relationships in the model differed across groups (Table 11). This multi-stage analytical strategy enables a comprehensive examination of how FDLS relates to SRL in flipped-classroom contexts, while also considering the roles of responsibility-related variables and DLSE.

**Table 7:** Regression of self-regulated learning (SRL) on family digital learning support (FDLS), controlling for age and gender (*N* = 550).

Predictor	B	SE	*t*	Significance
Constant	1.615	0.443	3.644	<0.001
Family digital learning support	0.504	0.038	13.260	<0.001
Age	−0.008	0.021	−0.363	0.717
Gender	0.038	0.053	0.721	0.471

*R*^2^ = 0.246.

**Table 8:** Baseline regression model predicting SRL from FDLS, RI, and HSLRR, with group as a covariate (*N* = 550).

Predictor	B	SE	*t*	*p*
Family digital learning support	0.248	0.039	6.316	<0.001
Responsibility internalization	0.353	0.038	9.208	<0.001
Home–school learning responsibility reconfiguration	0.220	0.037	5.980	<0.001
Group	0.073	0.047	1.555	0.120

*R*^2^ = 0.415.

**Table 9:** Full regression model predicting SRL from FDLS, HSLRR, RI, and DLSE (*N* = 550).

Predictor	B	SE	*t*	*p*
Constant	0.099	0.162	0.611	0.542
Family digital learning support	0.277	0.039	7.086	<0.001
Home–school learning responsibility reconfiguration	0.186	0.037	5.081	<0.001
Responsibility internalization	0.347	0.038	9.187	<0.001
Digital learning self-efficacy	0.155	0.035	4.430	<0.001

*R*^2^ = 0.433, Adjusted *R*^2^ = 0.428.

**Table 10:** Bootstrap mediation results (1,500 resamples) for the indirect effect of FDLS on SRL via DLSE (*N* = 550).

Indirect path	Effect	SE	95% CI
Family digital learning support → Digital learning self-efficacy → Self-regulated learning	−0.018	0.010	[−0.038, −0.001]

A 95% confidence interval excluding zero indicates statistical significance. Bootstrap analysis based on 1,500 resamples. The confidence interval does not include zero, indicating that the indirect effect is statistically significant.

**Table 11:** Moderation analysis testing FDLS × Group and RI × Group interactions predicting SRL (*N* = 550).

Predictor	Coef.	SE	*t*	Significance
Family digital learning support	0.392	0.056	6.980	< 0.001
Responsibility internalization	0.322	0.056	5.758	< 0.001
Group	0.110	0.270	0.408	0.683
Family digital learning support× group	0.024	0.077	0.308	0.758
Responsibility internalization × group	−0.035	0.077	−0.451	0.652

*R*^2^ = 0.324, Adjusted *R*^2^ = 0.318.

## 4 Results

### 4.1 Sample profile and descriptive properties of core constructs

Table 3 presents the descriptive statistics of the core study variables, including family digital learning support, home–school learning responsibility reconfiguration, responsibility internalization, digital learning self-efficacy, and self-regulated learning. The mean values of the variables are all close to the midpoint of the five-point scale, indicating moderate levels of perceived digital learning support, responsibility-related learning processes, and self-regulated learning among the participants. Specifically, the mean value of family digital learning support is 2.993 (SD = 0.694), suggesting that students generally report a moderate level of access to digital learning resources and supportive family learning conditions. Home–school learning responsibility reconfiguration (M = 2.990, SD = 0.723) and responsibility internalization (M = 2.999, SD = 0.693) also fall near the scale midpoint, indicating that students perceive a moderate degree of responsibility adjustment and internalization in flipped-classroom learning environments. Self-regulated learning shows a similar distribution (M = 2.989, SD = 0.709), suggesting that students’ reported ability to manage and regulate their learning processes remains at a moderate level across the sample. Digital learning self-efficacy (M = 3.004, SD = 0.667) likewise reflects a moderate level of perceived competence in handling digital learning tasks. The observed minimum and maximum values indicate that responses cover a substantial portion of the measurement scale, suggesting sufficient variability in students’ learning experiences and perceptions of digital learning conditions. Furthermore, the skewness and kurtosis values for all variables fall within acceptable ranges (|skew| < 1; |kurtosis| < 1), indicating that the distributions approximate normality and are suitable for subsequent correlation and regression analyses. Overall, these descriptive statistics suggest that the study variables demonstrate adequate variability and distributional properties, providing an appropriate basis for further statistical examination of the relationships among family digital learning support, responsibility-related learning processes, digital learning self-efficacy, and self-regulated learning.

### 4.2 Internal consistency and scale reliability of measurement constructs

Table 4 reports the internal consistency reliability and convergent validity of the measurement constructs used in this study. The Cronbach’s alpha coefficients indicate satisfactory internal consistency across all scales. Family digital learning support demonstrates good reliability (*α* = 0.870), indicating stable internal consistency among the items capturing the availability of digital learning resources and supportive family learning environments. Home–school learning responsibility reconfiguration (α = 0.863) and self-regulated learning (α = 0.886) also show strong reliability, suggesting that the items measuring perceived shifts in learning responsibility and students’ ability to regulate learning activities form coherent constructs. Responsibility internalization likewise demonstrates acceptable reliability (α = 0.819), indicating that the items consistently reflect students’ tendency to accept learning responsibilities as personal obligations. Digital learning self-efficacy shows a reliability coefficient of α = 0.714, which exceeds the commonly accepted threshold of 0.70 and therefore remains acceptable.

In addition to internal consistency reliability, CR values for all constructs range from 0.714 to 0.914, exceeding the recommended threshold of 0.70 and indicating adequate construct reliability. The AVE values for most constructs range from 0.533 to 0.640, exceeding the recommended value of 0.50 and supporting convergent validity. Although the AVE value for digital learning self-efficacy falls slightly below the conventional threshold (AVE = 0.384), the construct maintains acceptable composite reliability. The overall fit of the five-factor CFA model was acceptable (χ^2^(292) = 411.603, χ^2^/df = 1.41, CFI = 0.980, TLI = 0.977, RMSEA = 0.027, 90% CI [0.021, 0.033]), supporting the hypothesized measurement structure. Overall, most constructs demonstrated acceptable reliability and convergent validity. However, digital learning self-efficacy showed acceptable internal consistency but weaker convergent validity, as its AVE fell below the recommended threshold of 0.50, and findings involving this construct should therefore be interpreted with appropriate caution.

### 4.3 Zero-order associations among family digital learning support, responsibility-related processes, digital learning self-efficacy, and self-regulated learning

Table 5 presents the zero-order correlations among family digital learning support, home–school learning responsibility reconfiguration, responsibility internalization, digital learning self-efficacy, and self-regulated learning. The results indicate that family digital learning support is positively associated with home–school learning responsibility reconfiguration (r = 0.501, *p* < 0.001), suggesting that students who report higher levels of digital learning resources and supportive family learning conditions are also more likely to perceive a redistribution of learning responsibilities between classroom instruction and out-of-class learning. Family digital learning support also shows a positive correlation with self-regulated learning (r = 0.495, *p* < 0.001), indicating that students who receive stronger digital learning support from their family environments tend to report higher levels of learning regulation. In addition, home–school learning responsibility reconfiguration is positively associated with self-regulated learning (r = 0.561, *p* < 0.001), suggesting that students who recognize greater responsibility for managing their learning outside the classroom are more likely to demonstrate stronger self-regulated learning behaviors. Responsibility internalization also demonstrates positive correlations with family digital learning support (r = 0.324, *p* < 0.001), home–school learning responsibility reconfiguration (r = 0.365, *p* < 0.001), and self-regulated learning (r = 0.421, *p* < 0.001). These relationships indicate that supportive digital learning environments and perceived responsibility shifts are associated with stronger tendencies for students to internalize learning responsibilities as personal commitments. A somewhat different pattern emerges for digital learning self-efficacy. This variable shows a small negative correlation with family digital learning support (r = −0.085, *p* < 0.01) and no significant association with home–school learning responsibility reconfiguration (r = −0.030, ns), while weak positive correlations are observed with self-regulated learning (r = 0.161, *p* < 0.05) and responsibility internalization (r = 0.151, *p* < 0.05). Overall, the correlation structure suggests that family digital learning support is positively related to responsibility-related learning processes and self-regulated learning, while digital learning self-efficacy exhibits a more differentiated pattern of associations with the other study variables.

### 4.4 Common method bias test

To assess the potential influence of common method bias, Harman’s single-factor test was conducted using exploratory factor analysis. The results indicate that multiple factors were extracted from the measurement items, suggesting that the variance in the data is not dominated by a single underlying factor. Specifically, the first unrotated factor accounted for 31.046% of the total variance, which is below the commonly recommended threshold of 40%, indicating that a single factor does not explain the majority of the variance in the dataset. The variance explained by the subsequent factors was substantially smaller, with the first five factors accounting for 31.046, 10.506, 7.584, 6.315, and 5.572% of the variance, respectively. The distribution of variance across multiple factors suggests that the measurement structure is not substantially affected by a common latent source of variance. Therefore, the results provide preliminary evidence that common method bias is unlikely to pose a serious threat to the validity of the findings in this study, and the dataset is considered appropriate for subsequent correlation, regression, and mediation analyses.

### 4.5 Direct, indirect, and moderated associations with self-regulated learning

Table 7 presents the regression results examining the association between family digital learning support and self-regulated learning after controlling for demographic variables. The results indicate that family digital learning support remains a strong and statistically significant predictor of self-regulated learning (B = 0.504, t = 13.260, *p* < 0.001), suggesting that students who report greater access to digital learning resources and supportive family learning conditions tend to demonstrate higher levels of self-regulated learning. In contrast, the control variables do not reach statistical significance, with age showing no significant effect (t = −0.363, *p* = 0.717) and gender also remaining non-significant (t = 0.721, *p* = 0.471), indicating that demographic characteristics do not substantially explain variation in students’ self-regulated learning in this sample. Overall, the regression model explains 24.6% of the variance in self-regulated learning (*R*^2^ = 0.246), suggesting that family digital learning support represents an important contextual condition associated with students’ learning regulation. To further examine the joint influence of responsibility-related learning processes, a revised baseline regression model was estimated by including family digital learning support, home–school learning responsibility reconfiguration, and responsibility internalization as predictors while controlling for group differences (Table 8). The results show that all three learning-related variables display significant positive associations with self-regulated learning. Family digital learning support remains positively associated with self-regulated learning (B = 0.248, *p* < 0.001), responsibility internalization demonstrates the strongest positive association with self-regulated learning (B = 0.353, *p* < 0.001), and home–school learning responsibility reconfiguration is also significantly associated with self-regulated learning (B = 0.220, *p* < 0.001). In contrast, the grouping variable does not reach statistical significance (B = 0.073, *p* = 0.120), indicating that group membership does not independently explain variation in self-regulated learning once the core explanatory variables are taken into account. The revised baseline model explains 41.5% of the variance in self-regulated learning (*R*^2^ = 0.415). To further examine the role of digital learning self-efficacy, a full regression model was estimated by incorporating digital learning self-efficacy into the analysis while retaining family digital learning support, home–school learning responsibility reconfiguration, and responsibility internalization (Table 9). The results indicate that all four predictors remain significantly associated with self-regulated learning. Specifically, family digital learning support remains positively associated with self-regulated learning (B = 0.277, t = 7.086, *p* < 0.001), home–school learning responsibility reconfiguration also shows a significant positive effect (B = 0.186, t = 5.081, *p* < 0.001), responsibility internalization remains the strongest predictor in the model (B = 0.347, t = 9.187, *p* < 0.001), and digital learning self-efficacy shows a significant positive association with self-regulated learning (B = 0.155, t = 4.430, *p* < 0.001). The inclusion of digital learning self-efficacy increases the explanatory power of the model, with the full model explaining 43.3% of the variance in self-regulated learning (*R*^2^ = 0.433; Adjusted *R*^2^ = 0.428). Taken together, these findings suggest that family digital learning support is associated with students’ self-regulated learning both directly and in combination with responsibility-related learning processes and digital learning self-efficacy in flipped-classroom contexts.

### 4.6 Bootstrapped indirect effect of digital learning self-efficacy linking family digital learning support to self-regulated learning

**Figure 1:**
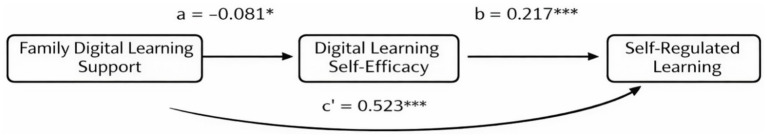
Mediation model of the indirect effect of family digital learning support (FDLS) on self-regulated learning (SRL) through digital learning self-efficacy (DLSE). Path coefficients are unstandardized estimates based on bootstrap analysis.

Table 10 and Figure 1 present the mediation results linking family digital learning support to self-regulated learning through digital learning self-efficacy. The bootstrap mediation analysis shows that family digital learning support is indirectly associated with self-regulated learning via digital learning self-efficacy, with an indirect effect of −0.018 (SE = 0.010) and a 95% confidence interval of [−0.038, −0.001]. Because the confidence interval does not include zero, the indirect effect is statistically significant. The results indicate a small but significant negative indirect pathway, suggesting that higher levels of family digital learning support are slightly associated with lower digital learning self-efficacy, while digital learning self-efficacy remains positively related to self-regulated learning. Overall, the findings indicate a small but statistically significant indirect association between FDLS and SRL through DLSE. However, because the indirect effect was negative and theoretically unexpected, it should be interpreted cautiously rather than as evidence of a robust mediation mechanism.

### 4.7 Moderation analysis of group effects

**Figure 2:**
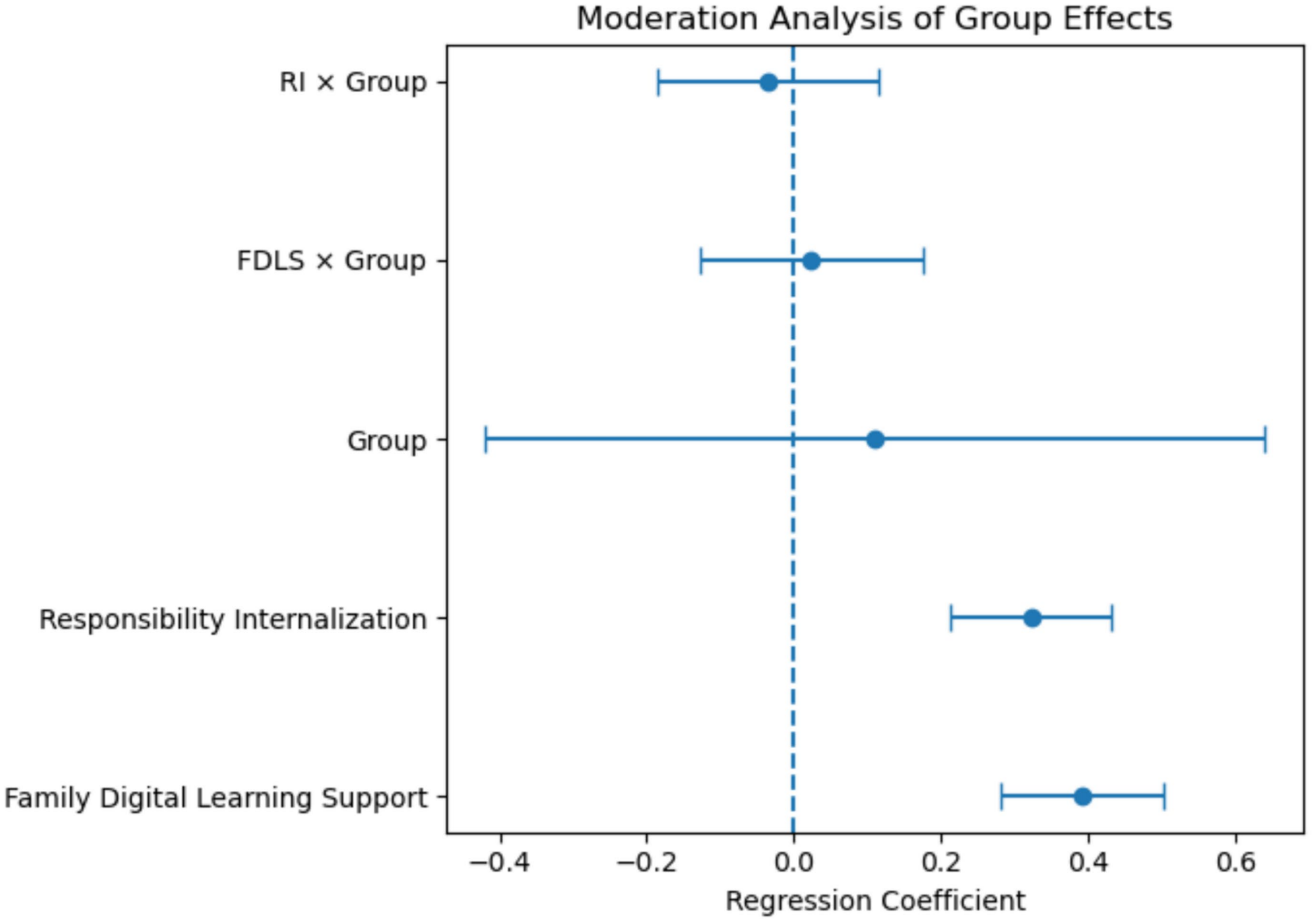
Unstandardized regression coefficients with 95% confidence intervals from the moderation model examining FDLS × Group and RI × Group interactions predicting self-regulated learning (SRL). Neither interaction term reached statistical significance (*R*^2^ = 0.324).

Table 11 and Figure 2 present the results of the moderation analysis examining whether the relationship between family digital learning support and self-regulated learning varies across groups. The regression model indicates that both family digital learning support and responsibility internalization remain significant predictors of self-regulated learning. Specifically, family digital learning support shows a positive association with self-regulated learning (B = 0.392, SE = 0.056, t = 6.980, *p* < 0.001), while responsibility internalization also demonstrates a significant positive effect (B = 0.322, SE = 0.056, t = 5.758, *p* < 0.001). These results suggest that students who report stronger digital learning support in their family environments and greater internalization of learning responsibility tend to exhibit higher levels of self-regulated learning. In contrast, the grouping variable itself does not reach statistical significance (B = 0.110, SE = 0.270, t = 0.408, *p* = 0.683), indicating that group membership does not independently explain variation in self-regulated learning. Furthermore, neither interaction term is statistically significant. The interaction between family digital learning support and group (B = 0.024, SE = 0.077, t = 0.308, *p* = 0.758) and the interaction between responsibility internalization and group (B = −0.035, SE = 0.077, t = −0.451, *p* = 0.652) both show coefficients close to zero. These results suggest that the relationships between the key predictors and self-regulated learning remain largely consistent across groups. In other words, the associations linking family digital learning support and responsibility internalization to students’ self-regulated learning do not substantially differ across the two group conditions. The overall model explains 32.4% of the variance in self-regulated learning (*R*^2^ = 0.324; Adjusted *R*^2^ = 0.318). Figure 2 visualizes the estimated regression coefficients and their confidence intervals, showing that the main predictors display clear positive effects while the interaction terms cluster around zero. Taken together, these findings indicate that the predictive role of family digital learning support and responsibility internalization in self-regulated learning appears to be relatively stable across different student groups, suggesting that the underlying mechanism operates in a broadly similar manner across the sample.

## 5 Discussion

### 5.1 Family digital learning support as a structural condition for sustaining out-of-class learning

The results of this study show that family digital learning support remains a significant predictor of self-regulated learning even after responsibility internalization and digital learning self-efficacy are included in the regression model (B = 0.277, *p* < 0.001). This finding suggests that digital learning conditions within the family environment function as an important structural support for sustaining students’ out-of-class learning activities. In addition to statistical significance, the magnitude of the observed effects indicates that family digital learning support explains a meaningful proportion of the variance in students’ self-regulated learning, highlighting the practical importance of the digital learning conditions available in students’ home environments. Flipped classroom models reorganize the learning process by shifting initial knowledge acquisition to the pre-class stage while reserving classroom time for discussion and higher-order learning activities (Bishop and Verleger, 2013). Such instructional arrangements implicitly assume that students are able to access digital learning materials and complete preparatory learning activities independently outside the classroom (Bergmann and Sams, 2012). The present findings support this assumption by showing that students who report stronger family digital learning support also demonstrate more stable self-regulated learning behaviors. Reliable access to digital devices and stable internet connectivity allows students to repeatedly engage with online learning materials and maintain relatively consistent study routines outside formal instructional settings (Borup et al., 2014). Under these conditions, students are more likely to develop planning, monitoring, and adjustment behaviors associated with self-regulated learning through repeated engagement with learning tasks. By contrast, when digital learning resources are limited or unstable, participation in pre-class learning activities may become fragmented, weakening the continuity of learning regulation even among students who are otherwise motivated to learn. From an ecological perspective, learning behaviors are shaped not only by instructional design but also by the environmental resources available within students’ broader learning contexts (Bronfenbrenner, 1979). In flipped classroom settings, the relocation of part of the learning process to home-based digital environments effectively extends the learning space beyond school boundaries, making family learning conditions increasingly relevant for students’ learning experiences. The findings therefore suggest that family digital learning support contributes to self-regulated learning primarily by stabilizing the material and temporal conditions required for sustained engagement rather than by directly increasing students’ motivation. This interpretation is consistent with research indicating that learning resources often influence engagement by shaping students’ opportunities to repeatedly practice learning strategies (Zimmerman, 2002). Continuous interaction with digital learning materials enables students to gradually establish stable study routines and strengthen the planning and monitoring behaviors that characterize self-regulated learning. At the same time, the findings highlight a structural issue in flipped classroom implementation. Although flipped learning is often presented as a pedagogical innovation designed to promote learner autonomy, its effectiveness partly depends on whether students possess adequate digital learning infrastructure within their home environments. When such conditions are unevenly distributed, the benefits of flipped classroom arrangements may also become uneven across students. These results therefore suggest that discussions of flipped classroom effectiveness should extend beyond classroom pedagogy and consider the broader digital learning environment within which students conduct out-of-class learning activities. In addition, the results showing a weak negative correlation but a positive regression association between family digital learning support and digital learning self-efficacy may reflect a potential suppression effect. When multiple related predictors are simultaneously included in the regression model, shared variance among predictors can alter the direction or magnitude of individual regression coefficients. This pattern suggests that digital learning self-efficacy may capture a distinct regulatory mechanism that becomes more visible once responsibility-related learning processes are statistically controlled.

### 5.2 Responsibility internalization as a key predictor and the cautious interpretation of the DLSE pathway

Responsibility internalization emerged as one of the most stable psychological factors associated with self-regulated learning in the present study. In the full regression model, responsibility internalization showed a strong positive association with self-regulated learning (B = 0.347, *p* < 0.001), suggesting that students who more strongly internalize learning responsibilities are more likely to demonstrate sustained self-regulated learning behaviors. This finding is consistent with the view that self-regulated learning depends not only on instructional design, but also on whether students come to regard learning tasks as personal commitments rather than externally imposed requirements. In digitally mediated and flipped learning environments, where a substantial portion of learning takes place outside direct teacher supervision, such internalization becomes particularly important because students must independently organize study schedules, monitor progress, and adjust strategies across different learning contexts. Previous research has likewise suggested that internalized responsibility plays an important role in transforming externally structured academic expectations into self-directed learning behaviors (Ryan and Deci, 2000). The present findings extend this perspective by suggesting that, in flipped-classroom contexts, supportive digital learning conditions may help create the practical basis upon which responsibility-oriented learning behaviors can be enacted more consistently. At the same time, digital learning self-efficacy also showed an independent positive association with self-regulated learning in the full model (B = 0.155, *p* < 0.001), indicating that students’ confidence in managing digital learning tasks remains a relevant complementary factor in learning regulation. However, the mediation analysis identified only a small negative indirect effect of family digital learning support on self-regulated learning through digital learning self-efficacy (indirect effect = −0.018, 95% CI [−0.038, −0.001]). Because this indirect pathway was weak in magnitude and theoretically unexpected in direction, it should be interpreted cautiously rather than as evidence of a robust mediation mechanism. One possible explanation is that the pattern reflects a suppression effect produced when conceptually related predictors are entered simultaneously into the model. Another possibility is that digital learning self-efficacy, as measured in this study, captures a more context-dependent form of perceived competence that does not increase linearly with stronger family support. Given these considerations, the more stable conclusion from the present results is that family digital learning support provides an important structural condition for out-of-class learning, while responsibility internalization serves as a more consistent psychological pathway associated with self-regulated learning. By comparison, the role of digital learning self-efficacy appears more limited and should be viewed as supplementary rather than central in the current model.

### 5.3 Contextual specificity of digital learning self-efficacy and cross-group stability of the mechanism

Digital learning self-efficacy displays a distinct pattern in the present study, suggesting that it reflects a more context-sensitive form of psychological regulation within digitally mediated learning environments. In the full regression model, digital learning self-efficacy remained positively associated with self-regulated learning (B = 0.155, *p* < 0.001), indicating that students’ confidence in managing digital learning tasks is still relevant to their perceived ability to regulate learning activities effectively. However, the correlation analysis revealed a more differentiated pattern. Digital learning self-efficacy showed relatively weak associations with several variables and even a small negative correlation with family digital learning support (r = −0.085, *p* < 0.01), suggesting that this construct does not simply increase alongside the availability of digital learning resources. Rather, digital learning self-efficacy may capture a situational form of perceived competence that becomes particularly relevant when students engage in digital learning tasks requiring independent problem solving and learning management. Prior research on self-efficacy similarly suggests that confidence in performing learning tasks often develops through repeated experiences of task engagement and self-evaluation rather than through external resource availability alone (Bandura, 1997). In this sense, digital learning self-efficacy may reflect students’ perceived competence in navigating digital learning platforms, organizing online learning activities, and sustaining engagement in technology-mediated learning environments. At the same time, the mediation analysis identified only a small negative indirect association between family digital learning support and self-regulated learning through digital learning self-efficacy (indirect effect = −0.018, 95% CI [−0.038, −0.001]). Because this indirect pathway was weak in magnitude and theoretically unexpected in direction, it should be interpreted cautiously rather than as evidence of a robust mediation mechanism. Moderation analysis showed that neither the main effect of group membership nor the interaction terms between family digital learning support, responsibility internalization, and group reached statistical significance, suggesting that the observed predictive patterns were broadly similar across the two groups. This result indicates that the positive roles of family digital learning support and responsibility internalization in self-regulated learning appear relatively stable across subgroup conditions, whereas the role of digital learning self-efficacy should be regarded as more limited and context-sensitive in the present model. Previous research has shown that core learning regulation processes often display structural stability across demographic contexts even when differences exist in learning resources or educational experiences (Pintrich, 2004). The present findings are broadly consistent with this perspective, but they also suggest that not all psychological variables operate with the same degree of stability or explanatory strength. Taken together, these results imply that while some core associations in the model remain relatively consistent across groups, the pathway involving digital learning self-efficacy should be interpreted more cautiously and should not be treated as a central explanatory mechanism in the current study.

### 5.4 Theoretical contributions and positioning within the existing literature

The present study makes several theoretical contributions to the flipped-classroom literature. Whereas prior research has largely examined family involvement, responsibility-related processes, and digital self-efficacy as separate lines of inquiry, the present study integrates these elements within a unified analytical framework. By simultaneously modeling family digital learning support, home–school learning responsibility reconfiguration, responsibility internalization, and digital learning self-efficacy as predictors of self-regulated learning, the study moves beyond single-factor explanations and offers a more comprehensive account of how structural conditions and psychological processes jointly shape learning regulation in flipped-classroom contexts. Building on this integrative approach, the study also introduces a conceptual distinction between home–school learning responsibility reconfiguration and responsibility internalization that has not been consistently maintained in previous work. Home–school learning responsibility reconfiguration captures students’ perceptions of how learning responsibilities are redistributed across classroom and out-of-class settings under flipped-classroom arrangements, whereas responsibility internalization refers to the extent to which students actively accept these redistributed responsibilities as personal obligations. Treating these as distinct constructs allows the study to differentiate between students’ awareness of structural responsibility shifts and their psychological ownership of learning tasks, a distinction that carries implications for how flipped-classroom courses are designed and how student engagement is supported. Beyond this conceptual refinement, the finding that family digital learning support exerts a direct and stable association with self-regulated learning even after psychological mediators are controlled further highlights the relevance of home-based material conditions in higher-education learning research. This extends the ecological perspective on learning (Bronfenbrenner, 1979) to digitally mediated university contexts, suggesting that the effectiveness of flipped-classroom instruction is partly contingent on conditions that lie outside the direct control of course designers and instructors. Taken together, these contributions suggest that theoretical accounts of self-regulated learning in flipped classrooms need to extend beyond instructional design variables and incorporate both the structural conditions of students’ home learning environments and the responsibility-related psychological processes through which those conditions shape learning behavior.

## 6 Limitations and conclusion

This study still has several boundaries that should be taken into account when interpreting the findings. First, the study employed a cross-sectional questionnaire design, so the relationships observed here are better understood as statistical associations grounded in theory rather than as strong causal effects. Second, all variables were measured through self-report instruments. Although Harman’s single-factor test suggested that common method bias was unlikely to pose a serious threat, the possibility of shared method influence cannot be completely ruled out. Third, although digital learning self-efficacy demonstrated acceptable internal consistency, its convergent validity was somewhat weaker than that of the other core constructs, which suggests that the indirect pathway involving this variable should be interpreted with appropriate caution. In particular, this study identified a small but statistically significant negative indirect effect of family digital learning support on self-regulated learning through digital learning self-efficacy, a finding that is somewhat counterintuitive from a theoretical perspective. A more appropriate interpretation is not to treat this result simply as evidence against the theoretical framework, but rather to regard it as a contextual finding that deserves further examination. On the one hand, it may reflect a suppression effect produced when conceptually related variables are entered into the model simultaneously. On the other hand, it may also suggest that digital learning self-efficacy captures a more context-dependent form of perceived competence that does not necessarily increase linearly with stronger family support. In this sense, readers are encouraged to view this finding dialectically: its statistical significance should be acknowledged, but its theoretical meaning should not be overstated. Overall, these limitations do not invalidate the main findings regarding the relationships among family digital learning conditions, responsibility internalization, and self-regulated learning in flipped-classroom contexts, but they do indicate that future research should further test these mechanisms through more refined measures, clearer grouping criteria, and longitudinal or structural equation modeling approaches.

Even though the study has certain limitations, the overall findings still suggest that self-regulated learning in higher-education flipped classrooms is shaped not only by instructional arrangements within the classroom but also by digital learning conditions in students’ home environments. Family digital learning support remained a stable predictor of self-regulated learning, indicating that access to digital devices, reliable internet connectivity, and appropriate home learning space plays a meaningful role in sustaining students’ out-of-class learning activities. Responsibility internalization also emerged as a strong and consistent explanatory factor, suggesting that when students actively take learning tasks as their own responsibility, they are more likely to develop sustained self-regulated learning behaviors. Digital learning self-efficacy showed an independent positive association in the full model, indicating that students’ confidence in managing digital learning tasks remains an important complementary factor in understanding learning regulation in flipped-classroom contexts. Although the indirect pathway involving digital learning self-efficacy showed some complexity, the overall findings still indicate that the effective implementation of flipped classrooms depends not only on redesigning classroom activities, but also on family digital conditions, the development of responsibility-related processes, and students’ ability to adapt to digitally mediated learning tasks. These results imply that promoting self-regulated learning in flipped classrooms may require not only improved instructional design and pre-class task organization, but also greater attention to whether students have adequate out-of-class digital learning conditions and whether course arrangements help them gradually internalize learning responsibility and strengthen learning management capacity. The study contributes to the flipped-classroom literature by extending explanations of self-regulated learning beyond classroom design alone and highlighting the joint relevance of family digital learning conditions and responsibility-related psychological processes.

## Data Availability

The raw data supporting the conclusions of this article will be made available by the authors, without undue reservation.
